# Effects of Inorganic Metabolites of Sulphate-Reducing Bacteria on the Corrosion of AZ31B and AZ63B Magnesium Alloy in 3.5 wt.% NaCl Solution

**DOI:** 10.3390/ma15062212

**Published:** 2022-03-17

**Authors:** Jinrong Li, Xin Liu, Jie Zhang, Ruiyong Zhang, Mingxing Wang, Wolfgang Sand, Jizhou Duan, Qingjun Zhu, Shenbao Zhai, Baorong Hou

**Affiliations:** 1 Key Laboratory of Marine Environmental Corrosion and Biofouling, Institute of Oceanology, Chinese Academy of Sciences, Qingdao 266071, China; lijinrrr@163.com (J.L.); wangmingxing21@mails.ucas.ac.cn (M.W.); duanjz@qdio.ac.cn (J.D.); zhuqingjun@qdio.ac.cn (Q.Z.); brhou@qdio.ac.cn (B.H.); 2 College of Materials Science and Engineering, Qingdao University of Science and Technology, Qingdao 266042, China; 3 Open Studio for Marine Corrosion and Protection, Pilot National Laboratory for Marine Science and Technology, Qingdao 266273, China; 4 Institute of Biosciences, University of Mining and Technology, 09599 Freiberg, Germany; wolfgang.sand@uni-due.de; 5 Department of Aquatic Biotechnology, University of Duisburg-Essen, 45141 Essen, Germany; 6 Textile Pollution Controlling Engineering Center of Ministry of Environmental Protection, College of Environmental Science and Engineering, Donghua University, Shanghai 200051, China; 7 Zibo Deyuan Metal Material Co., Ltd., Zibo 200051, China; bb1141403803@163.com

**Keywords:** magnesium alloy, sulphate-reducing bacteria, inorganic metabolites, electrochemical impedance spectroscopy, corrosion

## Abstract

This study seeks prevent and alleviate the failure of magnesium alloy anodes in pipelines, which we suspect is a problem related to SRB. The electrochemical corrosion behaviour of two kinds of magnesium alloys, AZ31B and AZ63B, in 3.5 wt.% NaCl solution with sulphide or phosphide—the two main inorganic metabolites of sulphate-reducing bacteria—were studied by electrochemical tests combined with other characterisation methods such as scanning electron microscopy and X-ray diffraction. The results show that the corrosion film formed by inorganic metabolites of SRB’s initial stage of corrosion (1–3 d) can lead to the corrosion of magnesium alloys. However, the loose and porous corrosion product film cannot protect the substrate effectively. The inorganic metabolites in the solution can accelerate the corrosion of the surface of magnesium alloy after the corrosion products have fallen off. This study provides a theoretical basis for alleviating the premature failure of magnesium alloy anodes and for corrosion protection in the future.

## 1. Introduction

There is a wide range of environments where microbiologically influenced corrosion (MIC) can occur. Due to the widespread presence of corrosive microorganisms, many metallic materials may be subjected to microbial corrosion [1,2]. Sulphate-reducing bacteria (SRB) account for more than 50% of all cases of microbiologically influenced corrosion [3]. The mechanisms of MIC caused by anaerobic microbes have been classified into two main types [4,5]. Type I MIC involves extra-cellular electron transfer (EET) and, thus, is also known as EET-MIC [6,7,8]. Secreted corrosive metabolites such as inorganic and organic acids can also cause MIC. Thus, they are also called Type II MIC or metabolite-MIC (M-MIC) [9]. However, it is still difficult to determine the leading factors of corrosion in the MIC processes because multiple species and their metabolites may be involved [10,11]. Thus, the influence of different microbial metabolites on the metal corrosion process has to be explored and clarified.

There are many metabolic modes of sulphate-reducing bacteria, and the anabolism of different species is also quite different. Some metabolites, common to various SRB, such as sulphide, organic acids, and oxidising agents, can be detected through various analysis methods [12]. These inorganic metabolites are mainly sulphide produced by microbial sulphate reduction. Some accelerate the corrosion of the matrix by reducing phosphate to phosphide on the metal surface. The phosphide causes significant corrosion [13,14].

Magnesium alloys are known as “the green engineering material of the 21st century”. Their density is approximately 1.7−2 g/cm^3^, which provides an excellent application performance as the lightest metallic structural material currently available. As a sacrificial anode material, magnesium alloys are very susceptible to corrosion due to their high chemical activity, which restricts their use on a large scale. Mg also has advantages due to high chemical activity, negative electrode potential, high driving voltage, and uniquely high specific strength. Because of the advantages mentioned above, magnesium anodes have been widely used for cathodic protection of buried pipelines. With the continuing development of offshore oil and gas installations, their corrosion problems have received considerably increased attention. The use of magnesium alloy anodes for cathodic protection is also an important research topic. However, in practical engineering applications, engineers often find that the current efficiency of magnesium anodes is too low. They often fail to achieve the desired protective effect and the danger of this is that it may result in failure. As this aspect of the research is needed, we hypothesize that the failure of the dissolution corrosion process of magnesium alloy was affected by sulphate-reducing bacteria.

There are two main reasons for the low corrosion resistance of magnesium alloys. First, the density coefficient of the oxide film formed naturally on the surface is less than 1, which makes it difficult to form a stable protection for the matrix. The second is the microgalvanic corrosion caused by the second phase and impurities [15,16]. The corrosion behaviour of a magnesium alloy is controlled through a slightly superficial protective layer [17]. Surface modification is a common method to enhance the corrosion resistance of magnesium alloys [18,19,20]. At present, although many studies have been performed on the surface corrosion of magnesium alloys, there are only a small number of reports concerning of the influence of SRB metabolites on magnesium alloy. Most studies have focused on the effects of SRB growth and sulphide production for the corrosion of carbon steel or copper [21,22]. For example, some studies discuss how extracellular polymeric substances (EPS) can protect Q235 steel from corrosion in the initial stage of the experiment, but its intrinsic inorganic metabolite sulphide will accelerate corrosion [23,24]. Therefore, it is difficult to determine the role of SRB metabolites for metal corrosion. Thus, the contribution effect of different metabolites to the corrosion process needs to be clarified.

The purpose of this study was to determine the influence of inorganic SRB metabolites—phosphorous compounds and sulphides—on the corrosion behaviour of magnesium alloys by means of electrochemical testing and surface morphology characterisation. The role of SRB inorganic metabolites on the corrosion properties of magnesium alloys is also discussed.

## 2. Materials and Methods

### 2.1. Preparation of Materials

The test materials were commercial 10 mm × 10 mm × 10 mm AZ63B and AZ31B sacrificial magnesium alloy anodes. The chemical compositions of the two alloys are shown in Table 1. A copper screw (Φ 3 mm) was inserted into one end of the magnesium alloy. After welding the screw with copper wire, the working face was exposed and the rest was sealed with an epoxy resin. According to national standard GB5776-86, the working face was ground step-by-step to 2000# with sandpaper. After ultrasonic cleaning, the working face was wiped with anhydrous ethanol. The samples were then rinsed with ultrapure water. All solutions were prepared using analytical reagents obtained from Shanghai Chemical Reagent Co. Ltd., (Shanghai, China). Ultrapure water was Millipore (18.2 MΩ-cm).

### 2.2. Experimental Medium

Synthetic seawater containing 3.5% NaCl was used as a blank control. According to the usual metabolism of SRB [25], Na_2_S·9H_2_O was used to study the effects of different concentrations of sulphur ion metabolism on magnesium alloys by preparing concentrations of 2, 4, and 6 mmol/L, respectively. Since phosphorous compounds are unstable, volatile, and highly toxic, they are not suitable for electrochemical tests. An appropriate concentration of PO_4_^3−^ was selected for electrochemical tests [26]. pH was between 6.0 and 6.5.

### 2.3. Electrochemical Corrosion Analyses

The working electrodes were AZ31B or AZ63B magnesium alloy coupons embedded in epoxy with a 10 mm × 10 mm exposed surface. A saturated calomel electrode (SCE) was used as the reference electrode. A 20 mm × 20 mm platinum plate served as the counter electrode for the three-electrode system. Electrochemical tests were carried out at room temperature. The working electrodes were immersed in the 3.5% NaCl solution with different concentrations of sulphides and phosphorous compounds for 14 days. Each test was started once the open-circuit potential of the system changed by less than 2 mV within 300 s. The excitation signal of the electrochemical impedance spectroscopy test was 10 mV sine wave voltage, and the scanning frequency range was 10 mHz~10^5^ Hz. The potential range of the polarisation curve scanning was relative open-circuit potential (±250) mV with a scanning speed of 0.5 mV/s.

### 2.4. Weight-Loss Testing

The two magnesium alloy samples were soaked in solutions of different concentrations and taken out after 6 days. Three parallel specimens were tested under each condition to ensure the reproducibility of the results. According to GB11112-89, the samples were pickled with boiling chromic acid solution for 1 min, then washed with distilled water immediately, cleaned in absolute ethanol, and dried using high-purity N_2_.

### 2.5. Surface Characterisation

In the process of immersion experiment, the white acicular product were falling off the electrode surface and sedimented at the bottom. At the end of the 14-day immersion, the precipitate was centrifuged and identified by using an X-ray diffractometer (XRD) after drying. After the experiment, the sample was washed with distilled water and then scrubbed with alcohol before drying. The electrode was removed and its surface morphology was visualised by scanning electron microscopy.

## 3. Results and Discussion

### 3.1. Open-Circuit Potential

**Figure 1:**
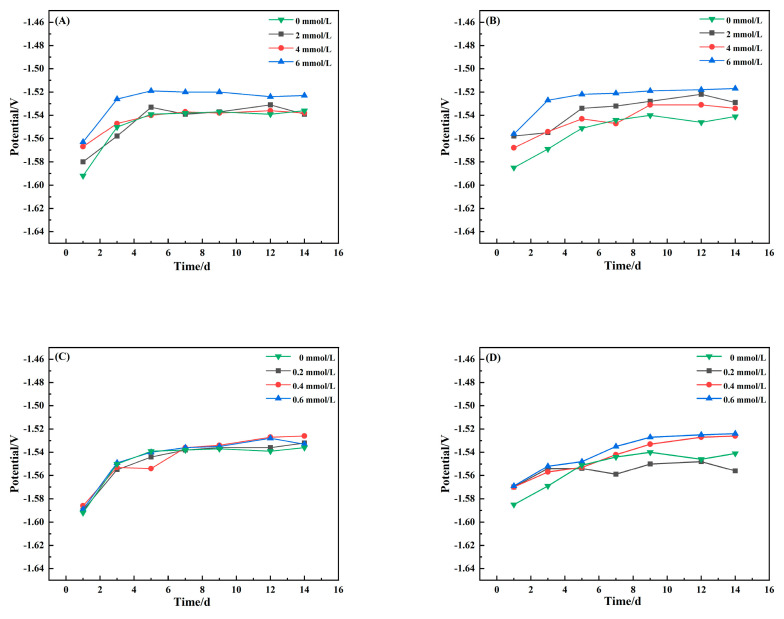
Open-circuit potential over time the magnesium alloys AZ31B (**A**,**C**) and AZ63B (**B**,**D**) in 3.5% NaCl solutions with different concentrations. Sulphide (**A**,**B**) and phosphate (**C**,**D**).

Figure 1A shows that the corrosion potential of the magnesium alloy AZ31B in the presence of different concentrations of sulphide is similar to that without addition of sulphide. The corrosion potential tends to be stable after a positive potential shift at the start. Figure 1B shows that the open-circuit potential of the magnesium alloy AZ63B samples at 6 mmol/L sulphide is always higher than that of the magnesium alloy at other concentrations. This indicates that more corrosion products are generated in the presence of 6 mmol/L sulphide. The accumulation of corrosion products on the surface of the sample causes some resistance to the continuous corrosion. The open-circuit potential of the sample soaked in 2 and 4 mmol/L S^2−^ solution increased from day 1 to day 5 and then stabilised.

Figure 1C shows that the open-circuit potential changes of sample AZ31B in PO_4_^3−^ solutions at different concentrations were roughly the same as those in the control assays (3.5% NaCl). The potential changed rapidly during the 1st–5th day Then, it became stable from day 7 to day 14. Data in Figure 1D show that the open-circuit potential of the magnesium alloy sample moved rapidly and then slowly for 1–3 d after immersion in 0.4 and 0.6 mmol/L phosphate solution. The results showed that the surface of the sample was covered by magnesium hydroxide hydrolysed by the corrosion products, which resulted in a decrease in the active reaction area and a positive potential shift [27,28].

### 3.2. Electrochemical Impedance Spectrum

**Figure 2:**
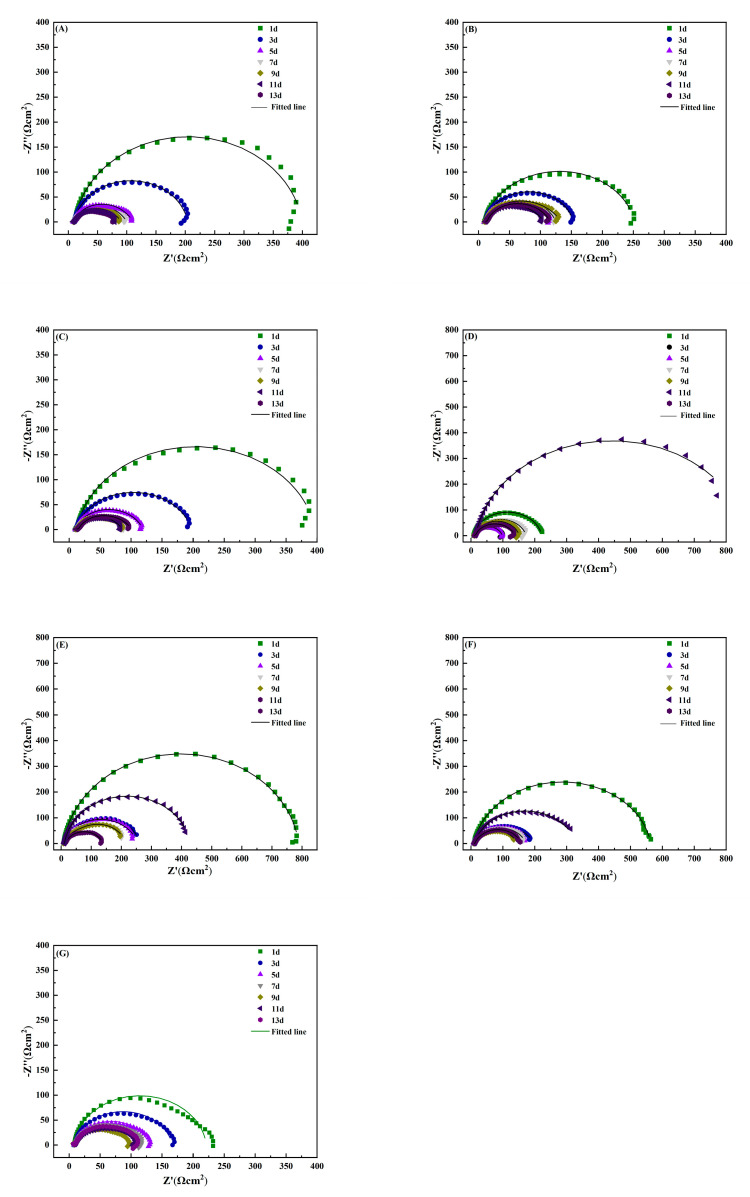
Electrochemical impedance spectra for AZ31B magnesium alloy immersed in 3.5% NaCl solution with different concentrations of simulated inorganic metabolites for different times. (**A**–**C**): soaked in 0.2, 0.4, and 0.6 mmol/L phosphate in 3.5% NaCl solution; (**D**–**F**): soaked in 3.5% NaCl solution with 2, 4, and 6 mmol/L sulphur ion concentration; (**G**): 3.5% NaCl solution.

Figure 2 shows the electrochemical impedance spectroscopy data of AZ31B soaked in 3.5% NaCl solution with different concentrations of sulphide over time. Studies show that the diameter of the capacitive arc in the Nyquist diagram is generally proportional to the corrosion resistance of electrode samples [29,30].

As can be seen in Figure 2A–C, with the increase in soaking time, the arc diameter of reactance for the AZ31B magnesium alloy in the phosphate solutions repeatedly experienced a process of gradually decreasing and then slightly increasing. This is due to the instability of the attachment of the corrosion products on the sample surface. The reaction resistance increased with the increase in the capacitance arc diameter. It is speculated that the corrosion products attached to the electrode surface cause an increase in the resistance of corrosion. Due to the shedding effects of corrosion products, part of the sample surface became exposed to the solution, which aggravated local corrosion. Consequently, the capacitive arc diameter was reduced. Compared with impedance diagrams for different concentrations, the arc reactance of the sample in phosphate solution of 0.4 mmol/L was significantly smaller than that of other concentrations. It indicates that the magnesium alloy is more susceptible to corrosion at a phosphate concentration of 0.4 mmol/L. As shown in Figure 2D–F, the magnesium alloy sample also exhibited a phenomenon of repeated arc changes for an addition of 3.5% NaCl solution with different concentrations of sulphide. This finding further proves that corrosion products of magnesium alloys are unstable. These adhere loosely to the substrate surface and cannot inhibit corrosion effectively.

**Figure 3:**
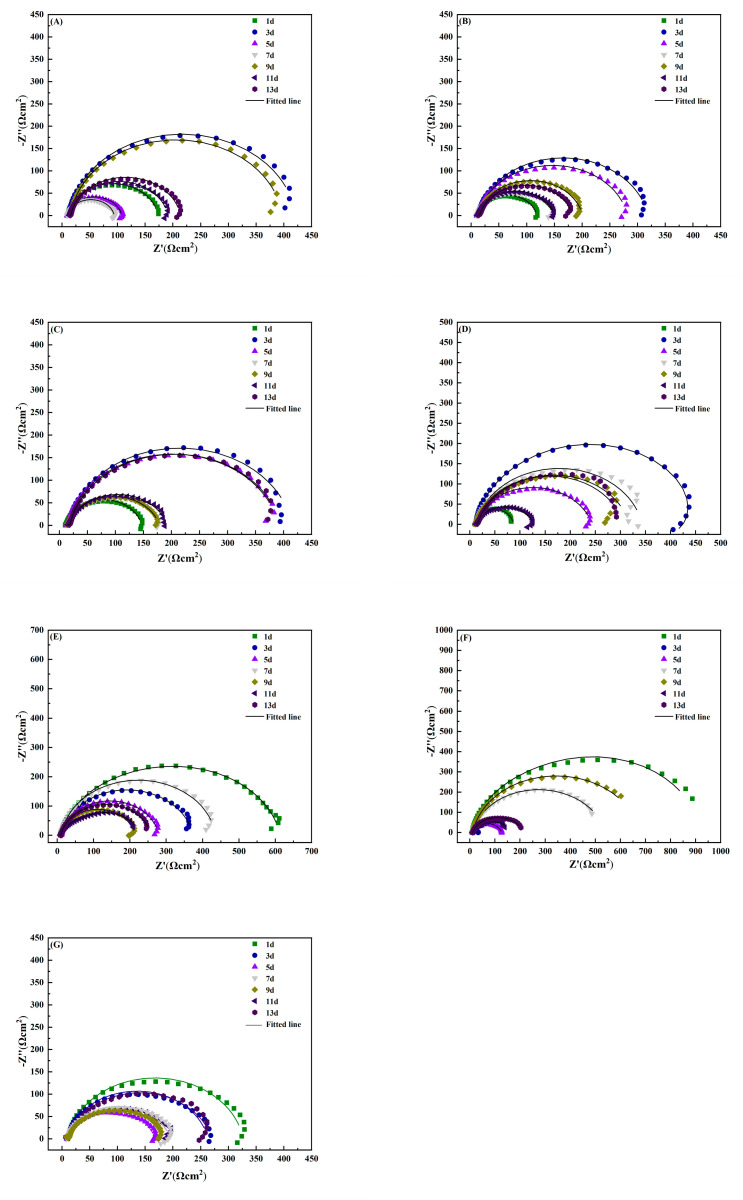
Electrochemical impedance spectra for AZ63B magnesium alloy soaked in 3.5% NaCl solution with different concentrations of simulated inorganic metabolites for different times. (**A**–**C**): soaked in 0.2, 0.4, and 0.6 mmol/L phosphate 3.5% NaCl solution; (**D**–**F**): soaked in 3.5% NaCl solution with 2, 4, and 6 mmol/L sulphur ion; (**G**): 3.5% NaCl solution.

Data from impedance spectra of AZ63B show that the surface was directly exposed to the solution with different concentrations of sulphide. With the progress of corrosion, the reactance arc gradually decreased and reached its minimum value on different days. Afterwards the radius of the capacitive arc increased with the increase in immersion time. This indicates that the corrosion rate decreased with an increase in surface corrosion products. If the corrosion product film did not fully cover the sample surface, the presence of sulphide accelerated the corrosion process. The higher the sulphide ion concentration was, the more easily corrosion products were enriched on the sample surface, as shown in Figure 3B. The arc radius of the capacitive reactance reached its maximum value on day 3, and then it reached the minimum value on day 7.

To summarise, the addition of sulphide or phosphate ions accelerates the corrosion process of magnesium alloys surface. The corrosion products formed by these ions have only a weak protection effect. This is probably related to the fact that they are loose and porous and can easily fall off the surfaces, as was observed by SEM.

**Figure 4:**
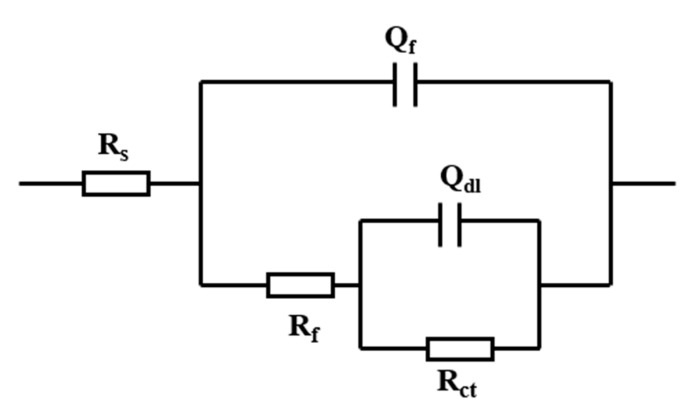
Schematic diagram of equivalent circuit derived from EIS results.

According to the experimental system, the equivalent circuit diagram shown in Figure 4 is selected for fitting and processing of EIS. R_s_ in the figure corresponds to solution resistance, R_f_ and Q_f_ correspond to the resistance and capacitance of the corrosion product film, respectively. R_ct_ and Q_dl_ correspond to charge transfer resistance and double layer capacitance, respectively. After fitting, the charge transfer resistance (Rct) values of magnesium alloy AZ31B sample and AZ63B in 3.5% NaCl solution with different concentrations of phosphate and sulphur ions were obtained. The values are shown in Table 2, Table 3, Table 4 and Table 5. It can be seen that R_ct_ is not a constant value in the experiment, which also indicates the instability of the corrosion product film on the magnesium alloy surface. R_ct_ values for the magnesium alloy change the most if the sulphide ion concentration is 6 mmol/L (Table 2). In Table 3, the magnesium alloy sample values have a minimum R_ct_ value when the phosphate concentration is 0.4 mmol/L.

### 3.3. Characterisation of Electrochemical Polarisation Curves

**Figure 5:**
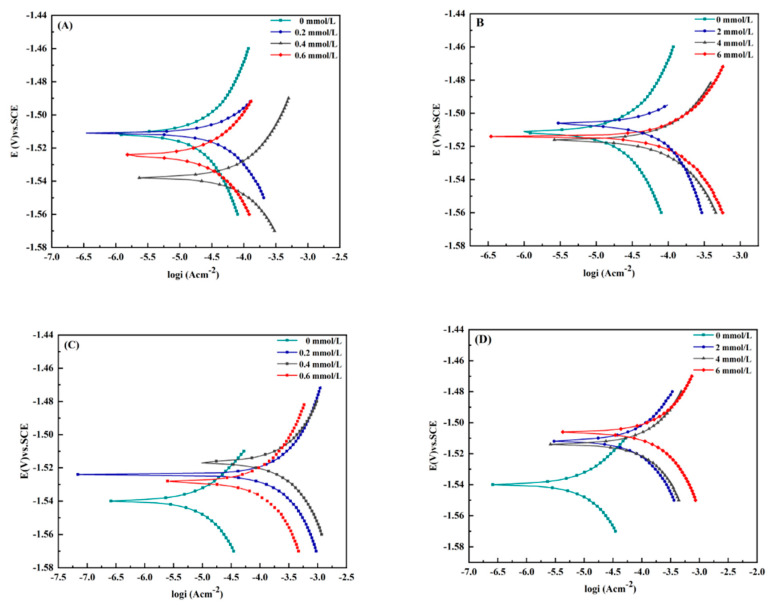
Potentiodynamic polarisation curves of magnesium alloys soaked in 3.5% NaCl solution with different concentrations of inorganic metabolites for 14 d. (**A**,**B**): magnesium alloy AZ31B in phosphate and sulphur ion solution; (**C**,**D**): AZ63B magnesium alloy in solution with phosphate and sulphur ions.

According to Faraday’s second law, the corrosion current density is proportional to the corrosion rate [31]. After 14 days of immersion, the potentiodynamic polarisation curve was recorded. Table 6 lists the Tafel parameter values determined from Figure 5. The results show that the corrosion current densities of the magnesium alloys increase if immersed in solutions with different concentrations of inorganic metabolites. The maximum corrosion current density of the two magnesium alloy samples reached 0.4 mmol/L solution and also 6 mmol/L sulphide. Both, *β_a_* and *β_c_* were greater than 1, which indicates that the corrosion process is controlled by anodic polarisation.

### 3.4. Weight Loss Data Analysis

Figure 5 shows the corrosion rates of the magnesium alloy calculated from weight-loss measurements after 6 days of testing under varied concentrations. The corrosion rate can be calculated as follows:

$$\nu =8.76\times \frac{w_{0}-w_{1}}{AT\rho }$$

where ν is the average corrosion rate of the sample (mm/a), *w*_0_* − w*_1_ is the weight lost after removal of corrosion products (g), *A* is the sample area (m^2^), *T* is the test cycle, and *ρ* is the density of magnesium alloy (1.78 g/cm^3^).

It is seen that the polarisation curves are in good agreement with the corrosion rate of magnesium alloy measured by weight loss. When the phosphate concentration is 0.4 mmol/L and sulphide ion concentration is 6 mmol/L, the corrosion rate is the most significant.

### 3.5. Corrosion Product Analysis

**Figure 6:**
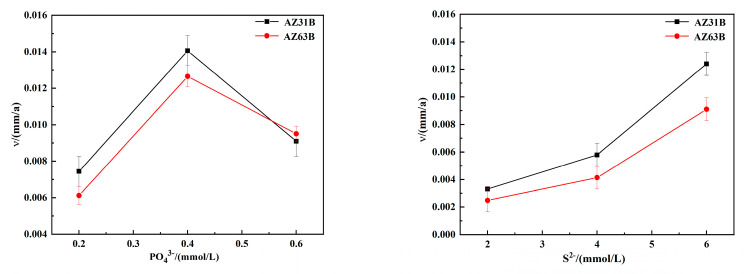
Corrosion rates of the magnesium alloy calculated from weight-loss measurements after 6 days of testing under varied concentrations.

Typical magnesium alloy corrosion products in electrolyte solution with two kinds of inorganic metabolites were observed by SEM. Figure 6 shows the micromorphology of two magnesium alloys immersed in solutions with different inorganic metabolites. The surface morphology of the corrosion products showed great differences.

**Figure 7:**
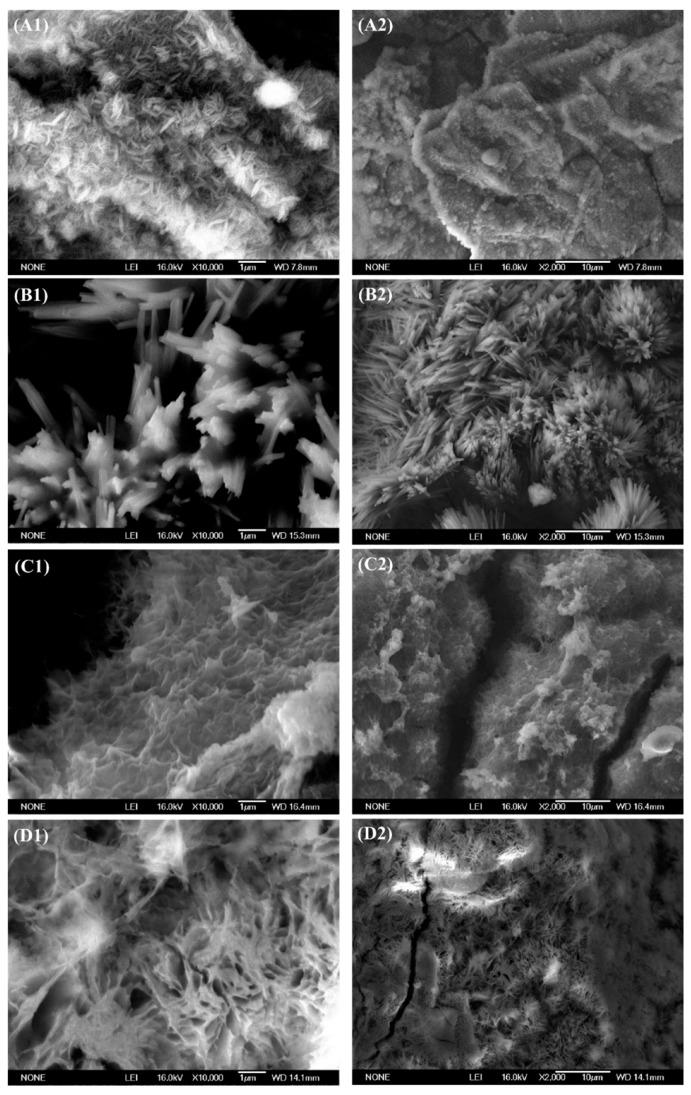
Influence of different inorganic metabolites of SRB on the morphology of magnesium alloys observed by SEM. (**A1**,**A2**): alloy AZ31B immersed in 2 mmol sulphide ions, (**B1**,**B2**): alloy AZ63B immersed in 2 mmol sulphide ions, (**C1**,**C2**): alloy AZ31B immersed in 0.4 mmol phosphate ions, and (**D1**,**D2**): alloy AZ63B immersed in 0.4 mmol phosphate ions.

Figure 7A shows that—if sulphides were added to the simulated seawater solution—the corrosion layer of the magnesium alloy AZ31B is present mainly as needle-like products of corrosion (Figure 7(A1)). The corrosion sediments are accumulated loosely in large spongy chunks. Additionally, some microcracks or voids were also visible (Figure 7(A2)). From Figure 7B it can be seen that the corrosion products on the surface of the magnesium alloy AZ63B are loose and needle-like. If phosphate was added to the solution, as can been seen in the images in Figure 7C,D, then the corrosion products on the sample surface formed loose and porous layers. They were more porous than those produced when sulphur ions had been added. Therefore, a local corrosion of the metal matrix was more likely to occur, which was consistent with the results shown in Table 4. Similarly, the loose and porous state of the corrosion products is consistent with the experimental results of XRD.

The appearance of the surface aggregation state of corrosion products indicates that the corrosion product film cannot prevent contact between the film and the surface of the magnesium alloys. Some loose corrosion products cannot adhere to the surface of the metal and easily fall off. This may be accompanied by the magnesium alloy metal particles falling off as well. The results show that the corrosion product layer cannot permanently protect the magnesium alloy during an immersion test.

**Figure 8:**
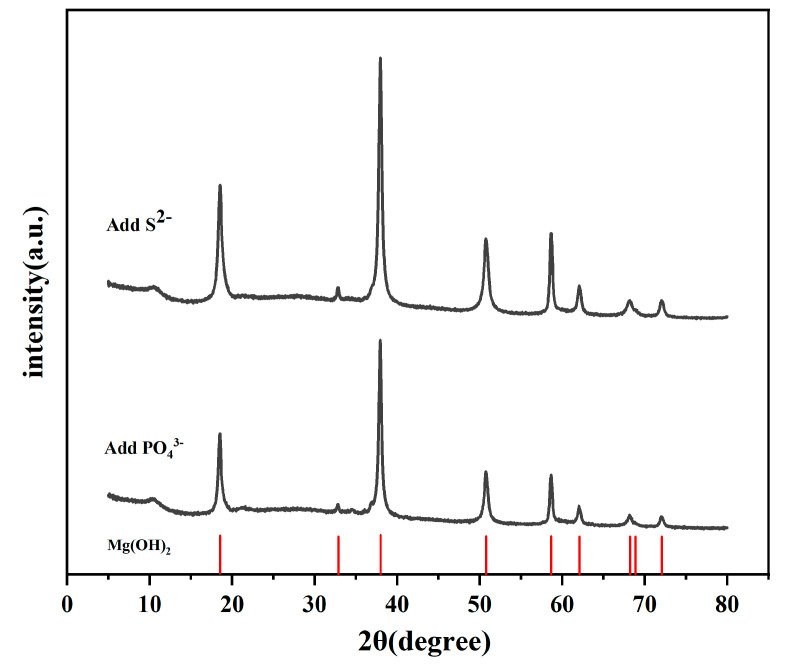
XRD patterns of the precipitates in 3.5%NaCl solution containing 0.4 mmol/L PO_4_^3−^ and 4 mmol/L S^2−^ after 14 days and the corresponding standard peaks.

The white precipitate at the bottom of the beaker during the immersion experiment was tested by X-ray diffractometry. The precipitate showed a good antipodicity Mg (OH)_2_ standard (Figure 8). The only difference is the peak strength, which probably is related to the crystallinity of the corrosion products. This indicates that the corrosion products generated during the experiment react with water and then fall off from the substrate surface in the form of Mg (OH)_2_. If the magnesium alloy is in contact with the corrosive medium, the area without oxidation film covered can react preferentially as an anode and then corrode:

Mg → Mg^2+^ + 2e^−^ (1)

Under the action of electromigration, the corrosive medium moves to the pit and further promotes the dissolution of magnesium. At the same time, magnesium ions undergo a hydrolysis reaction:

Mg^2+^ + 2H_2_O → Mg (OH)_2_ + 2H^+^ (2)

The generated H^+^ accumulate continuously, which improves the acidity in the etching pit. At the same time, acidic conditions interacting with the magnesium metal cause an autocatalytic reaction to take place.

The corrosion products may accumulate and block the corrosion hole, thus preventing an ion exchange between the environment in the hole and outside the medium, which blocks the battery and aggravates corrosion further [32]. Therefore, all precipitates obtained by XRD are Mg(OH)_2_.

## 4. Conclusions

The following preliminarily conclusions can be drawn:

1. A certain amount of SRB inorganic metabolites can accelerate the corrosion of magnesium alloys AZ31B and AZ63B. If the phosphate concentration is 0.4 mmol/L or the sulphide ion concentration is 6 mmol/L, the corrosion is most significant.
2. At the beginning of the immersion test, the corrosion product film can prevent corrosion from continuing. However, it was not stable. About 1–3 d after the experiment, the corrosion products start to fall off from the surface of the specimen. Even the magnesium blocks on the metal surface of the sample may fall off, thus accelerating corrosion. Over time, the corrosion products build up on the metal substrate surface again; however, they cannot provide effective protection.
3. The corrosion product layer attached to the surface is loose and accumulates microcracks and voids. This layer does not provide long-term and effective protection against corrosion.

Free SRB metabolites cannot form dense and protective oxidation films on the surface of the two magnesium alloys. The experimental results show that the corrosion process is similar for both magnesium alloys. Loose and porous corrosion products accelerate the corrosion process. The effect of the inorganic metabolites on corrosion of two magnesium alloys was elucidated. The results provide a theoretical basis for exploring the key factors of SRB metabolites restricting or accelerating the performance changes of magnesium alloy anodes.

## Figures and Tables

**Table 1:** The chemical composition of two Mg alloy sacrificial anodes (wt.%).

Composition	Al	Be	Si	Ca	Zn	Mn	Cu	Fe	Ce	Mg
AZ31B	3.19	0.100	0.020	0.040	0.810	0.334	0.050	0.005	—	95.5
AZ63B	5.30	—	—	—	2.50	0.150	—	—	trace	92.1

—: no detection or below detection limit.

**Table 2:** R_ct_ values obtained by EIS fitting of magnesium alloy AZ31B immersed in 3.5% NaCl solution with different concentrations of S^2^^−^.

Immersion Time (d)	2 mmol·L^−1^	4 mmol·L^−1^	6 mmol·L^−1^
1	201.8	764.4	554.2
3	83.30	249.6	203.8
5	83.16	241.3	160.8
7	157.7	47.21	159.6
9	138.0	41.43	132.7
11	409.8	346.5	312.3
13	121.4	123.1	144.4

**Table 3:** R_ct_ values obtained by EIS fitting of magnesium alloy AZ63B immersed in 3.5% NaCl solution with different concentrations of S^2−^.

Immersion Time (d)	2 mmol·L^−1^	4 mmol·L^−1^	6 mmol·L^−1^
1	62.63	605.5	885.9
3	430.4	358.8	39.94
5	227.5	268.8	113.9
7	329.3	422.4	526.1
9	292.5	201.5	662.1
11	110.4	60.71	149.8
13	301.2	246.0	198.6

**Table 4:** R_ct_ values obtained by EIS fitting of magnesium alloy AZ31B immersed in 3.5% NaCl solution with different concentrations of PO_4_^3−^.

Immersion Time (d)	0.2 mmol·L^−1^	0.4 mmol·L^−1^	0.6 mmol·L^−1^
1	389.4	219.0	381.8
3	191.4	140.9	184.8
5	96.47	102.3	104.9
7	83.56	112.8	74.18
9	73.56	118.4	74.90
11	63.05	89.03	70.85
13	58.01	99.82	84.81

**Table 5:** R_ct_ values obtained by EIS fitting of magnesium alloy AZ63B immersed in 3.5% NaCl solution with different concentrations of PO_4_^3−^.

Immersion Time (d)	0.2 mmol·L^−1^	0.4 mmol·L^−1^	0.6 mmol·L^−1^
1	158.6	105.0	133.6
3	381.3	305.6	399.4
5	93.51	268.2	375.9
7	79.29	134.8	163.1
9	398.5	185.4	163.3
11	192.0	135.1	175.3
13	197.0	165.7	371.0

**Table 6:** Potential polarisation curve parameters for magnesium alloy immersed in different solutions for 14 days.

Magnesium Alloy	PO_4_^3−^ (mmol/L)	S^2−^ (mmol/L)	i_corr_ (Acm^−2^)	E_corr_ (V) vs. SCE	β_a_(mv/Decade)	β_c_(mv/Decade)
AZ31B	0.0	0.0	2.90 × 10^−5^	−1.513	94.32	−91.18
	0.2		4.26 × 10^−5^	−1.509	38.94	−36.86
	0.4		1.19 × 10^−4^	−1.538	52.11	−43.47
	0.6		4.02 × 10^−5^	−1.522	60.95	−49.70
		2.0	7.27 × 10^−5^	−1.506	24.91	−24.70
		4.0	5.85 × 10^−5^	−1.515	35.86	−33.26
		6.0	1.25 × 10^−4^	−1.514	52.21	−47.00
AZ63B	0	0	1.06 × 10^−5^	−1.539	48.90	−45.90
	0.2		2.57 × 10^−4^	−1.524	47.16	−45.40
	0.4		3.96 × 10^−4^	−1.515	52.84	−50.14
	0.6		1.72 × 10^−4^	−1.526	55.41	−54.25
		2.0	1.31 × 10^−4^	−1.512	44.07	−39.01
		4.0	1.75 × 10^−4^	−1.515	59.54	−55.75
		6.0	2.80 × 10^−4^	−1.505	55.94	−49.14

## Data Availability

All the data are already provided in the main manuscript. Contact the corresponding author if further explanation is required.
